# Stream food webs in tropical mountains rely on allochthonous carbon regardless of land use

**DOI:** 10.1371/journal.pone.0295738

**Published:** 2023-12-15

**Authors:** Alonso Ramírez, Gabriela Vázquez, Vinicio Sosa, Pavel García, Gonzalo Castillo, José García-Franco, Ma. Luisa Martínez, Klaus Mehltreter, Eduardo Pineda, M. Susana Alvarado-Barrientos, Federico Escobar, Carolina Valdespino, Adolfo Campos

**Affiliations:** 1 Dept. Applied Ecology, North Carolina State University, Raleigh, NC, United States of America; 2 Instituto de Ecología, A.C., Xalapa, Veracruz, México; 3 Escuela de Biología, Universidad de San Carlos de Guatemala, Cdad. de Guatemala, Guatemala; 4 Ecology and Evolution Program, University of Montana, Missoula, MT, United States of America; University of Eldoret, KENYA

## Abstract

The relative importance of allochthonous and autochthonous carbon (C) as sources of energy for tropical stream food webs remains an open question. Allochthonous C might be the main energy source for small and shaded forest streams, while autochthonous C is more likely to fuel food webs draining land uses with less dense vegetation. We studied food webs in cloud forest streams draining watersheds with forests, coffee plantations, and pastures. Our goal was to assess the effects of those land uses on the C source and structure of stream food webs. The study took place in tropical montane streams in La Antigua Watershed, in eastern Mexico. We selected three streams per land use and sampled biofilm and leaf litter as the main food resources, and macroinvertebrates and aquatic vertebrates from different trophic guilds. Samples were analyzed for δ^13^C and δ^15^N isotopes. Using a Bayesian mixing model, we estimated the proportional assimilation of autochthonous and allochthonous carbon by each guild. We found that consumers were mostly using allochthonous C in all streams, regardless of watershed land use. Our findings indicate that montane cloud forest streams are dominated by allochthony even in watersheds dominated by pastures. Abundant precipitation in this life zone might facilitate the movement of allochthonous C into streams. While food webs of streams from coffee plantations and pastures also rely on allochthonous resources, other impacts do result in important changes in stream functioning.

## Introduction

Stream food webs rely on two major sources of C, allochthonous inputs from terrestrial vegetation (e.g., leaf litter) and autochthonous inputs of algal growth in the channel [1]. The type and amount of riparian vegetation and catchment land use determines the amount and quality of resources available for stream food webs. In most temperate regions, inputs of riparian C are the main energy source for small forest stream food webs [2]. Shading from riparian vegetation limits algal growth, reducing inputs of autochthonous C and multiple trophic levels are linked to allochthonous C [3]. Studies in tropical streams are less definitive, with some authors arguing for a larger role of algal C as the main source of energy for tropical food webs [e.g., 4, 5]. Studies combining measures of food consumption with stream metabolism indicate that the overall pattern could be similar to that described for temperate regions [6]. Interestingly, studies in small tropical streams in Brazil indicate that the amount of primary productivity in forested streams is high enough to fuel aquatic food webs, but most consumers rely on allochthonous C [7].

Removal of riparian or catchment vegetation changes the proportions of allochthonous and autochthonous C available to consumers, severely altering stream food webs. Land uses that decrease canopy cover and reduce shading over the stream (e.g., pastures, logging) could increase algal production and decrease vertical inputs of leaf litter [8]. Stream food webs respond to those changes by switching consumption from allochthonous to autochthonous C. This response has been documented in temperate regions [9] and a similar trend has been suggested for tropical regions, at least for some consumers (benthic macroinvertebrates [10]). Extreme land use changes (e.g., conversion of forest into pasture) cause the largest changes in stream food webs. Other land use changes (e.g., those that retain wide strips of riparian forest) have less dramatic effects. In tropical regions, less intense agricultural activities often replace riparian vegetation with other shrubs. Coffee plantations, for example, tend to leave no native vegetation at the stream edge, but coffee plants (and shade trees when present) shade stream channels and provide leaf litter for consumers [11].

Tropical montane cloud forest is a unique and valuable ecosystem that is changing rapidly due to human activities and land use conversion [12]. Cloud forests are restricted to the upper parts of tropical mountain ranges, supporting high levels of endemism [13, 14] and rich hydrological resources [15]. They are a conservation priority for the protection of biodiversity and their rich hydrological resources [14, 16]. Land use conversion, from forest to agriculture (e.g., coffee) and pastures, along with a changing climate that might reduce cloud cover, are major anthropogenic impacts affecting this region [15, 17]. Cloud forests provide a unique opportunity to understand how land use changes affect stream food webs by altering their energy basis. Forest streams are often heavily shaded by riparian vegetation and experience a low light environment, with frequent cloud cover further limiting light penetration [18]. Conversion into pastures drastically changes the light environment, potentially increasing algal C availability, while other land uses could create less drastic changes. Shade coffee plantations often reach stream margins and maintain low light levels, but impact streams by increasing erosion and consequently sediment, nutrient, and agrochemical inputs [18].

In this study, we focused on cloud forest streams draining watersheds with three dominant land uses that are common in many montane tropical regions: forests, coffee plantations, and pastures. Our goal was to assess the effects of those land uses on aquatic food webs and their main C sources in tropical montane streams in the La Antigua Watershed, Mexico. We hypothesized that the main C source is driven by the availability of autochthonous C. We predicted that forest streams would rely on allochthonous inputs of C as predicted for small forest headwater streams in general. Pasture streams were expected to have high algal growth and food webs based on autochthonous C. Coffee plantation streams should fall in between, but would be expected to be more similar to forest streams, because they are shaded and structurally alike to forest.

## Materials and methods

### Ethics

Our study did not involve endangered or protected species. Vertebrate sampling was conducted under sampling permit FAUT-0303 from Secretaría de Medio Ambiente y Recursos Naturales (SEMARNAT), Mexico.

### Study sites

**Fig 1:**
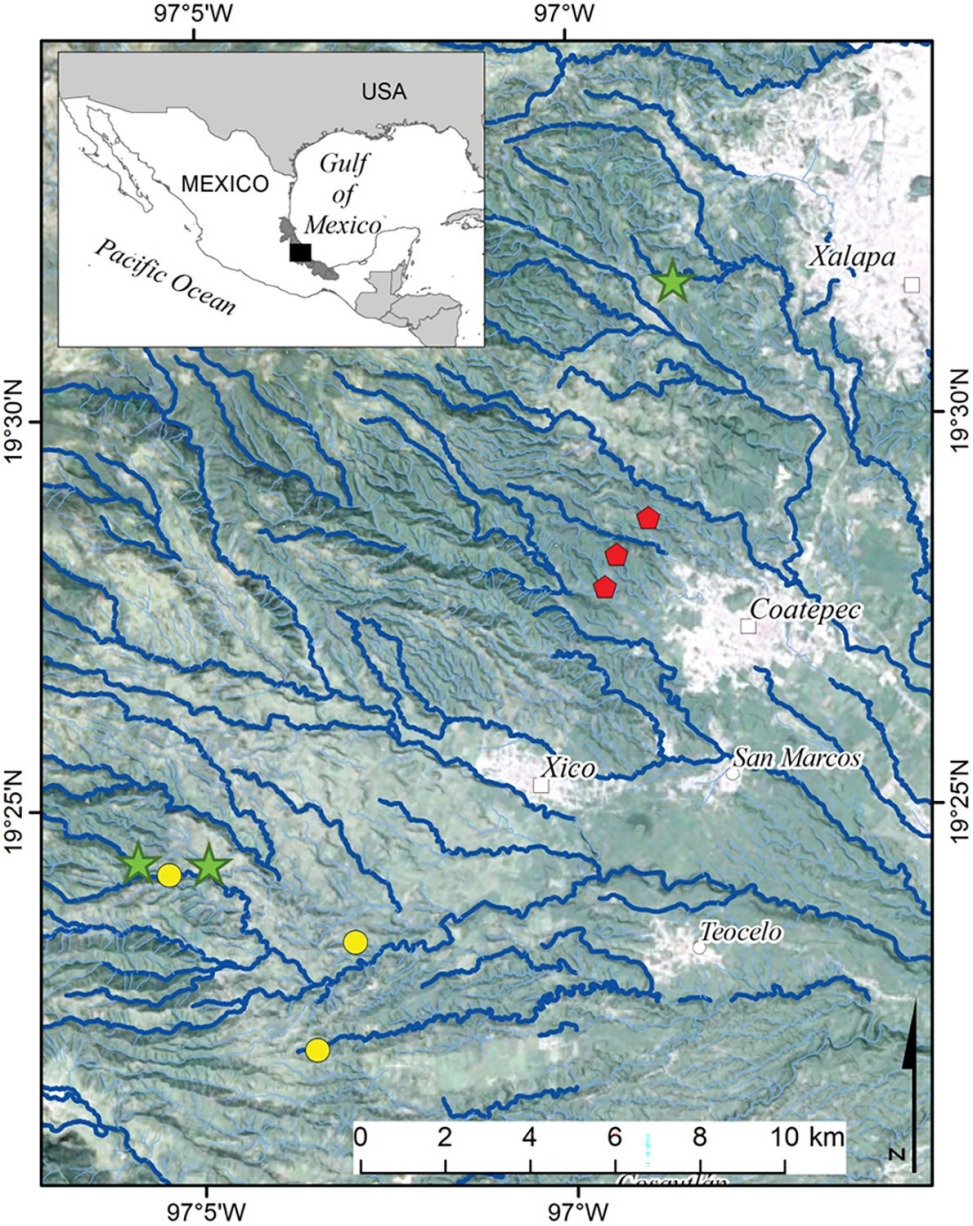
Sampling sites in the upper La Antigua watershed, Veracruz, Mexico. The nine study streams were first or second order draining forest (green stars), coffee plantations (red pentagons), and pastures (yellow circles). Since we selected first to second order streams, not all symbols fall on a visible stream channel. Figure modified from Vázquez et al. (2023).

Our study was conducted in streams draining Tropical Mountain Cloud Forest (TMCF) (between 1500 and 2000 m a.s.l.) in the La Antigua watershed (Fig 1). La Antigua is a 2,623 km^2^ basin that drains part of the states of Puebla and Veracruz (19°050’ and 19°340’N; 96°060’ and 97°160’W) in eastern Mexico, discharging into the Gulf of Mexico [19]. Temperature and precipitation vary with elevation; annual means are 22°C and 1300 mm at 600 m a.s.l. and 12°C and 2000 mm at 2500 m a.s.l. [20]. This watershed is an area of high biodiversity and hydrological importance [21]. TMCF is the most biodiverse life zone in Mexico [22, 23]. Land use comprises a mixture of forest (mostly restricted to steep slopes), agricultural fields, shade coffee plantations, pastures, and urban areas [17].

We selected nine first or second order streams that are part of our ongoing research program in TMCF. At each stream, we delimited a 100-m reach that was dominated by one of the three study land uses: forests, coffee plantations, and pastures (Fig 1). Watersheds contained a mixture of land uses, but our selected reaches had at least a 100-m wide strip of the particular land use on each side. Forest streams had dense riparian vegetation of either natural forest or secondary growth. Coffee plantations were interspersed with trees (e.g., shaded coffee plantations), with the plantation often reaching the stream margin. Pastures were for cattle grazing and some riparian vegetation was present (e.g., scattered riparian trees). Stream physical and chemical characterization and aquatic organisms sampling were conducted within the 100 m study reach (Table 1).

**Table 1:** Physicochemical characteristics of the study streams, grouped by land use. Values are means, standard deviations (SD), and minimum-maximum (Min-Max) values for the three streams sampled per land use type. Lines in bold represent significant differences among land uses, means with different letters are significantly different from the others.

Variable	Forest			Coffee plantation			Pasture		
	Mean	SD	Min-Max	Mean	SD	Min-Max	Mean	SD	Min-Max
Channel Width (m)	3.3	1.3	1.8–3.9	2.8	0.9	2.0–3.9	3.2	2.0	1.4–5.4
Channel Slope (%)	7.0	1.7	5–8	12.0	6.0	6.0–18.0	7.3	3.8	3.0–10.0
Canopy cover (%)	82.3	9.5	73.0–92.0	69.7	7.7	63.0-78-0	54.6	20.2	43.0-78-0
Temperature (°C)	14.6	1.3	13.4–16.3	17.1	1.0	15.7–18.0	14.9	2.8	9.9–17.8
Dissolved Oxygen (mg/L)	6.4	0.3	5.9–6.7	6.2	0.1	6.0–6.4	5.8	0.6	5.1–6.5
Conductivity (μS/cm)	32.2	8.4	21.0–39.3	26.2	1.3	24.3–27.6	24.8	12.9	13.0–41.3
pH	7.0	0.2	6.7–7.2	6.6	0.4	6.1–7.1	6.6	0.6	5.8–7.4
**Total Suspended Solids (mg/L)**	**5.3** ^**a**^	**6.1**	**1.8–17.6**	**22.1** ^**b**^	**21.2**	**5.8–60.6**	**13.0** ^**ab**^	**17.5**	**2.2–46.9**
Alkalinity (mg/L)	23.3	5.6	17.0–30.3	16.0	0.7	15.4–17.3	18.9	9.6	12.8–32.2
**N-NH**_**4**_ **(μM)**	**3.3** ^**a**^	**4.9**	**0–10.9**	**2.7** ^**a**^	**2.0**	**0–5.0**	**0.1** ^**b**^	**0.2**	**0.0–0.6**
N-NO_3_ (μM)	14.7	3.4	10.9–19.6	10.7	3.6	7.0–15.7	10.4	6.7	3.7–19.0
N-NO_2_ (μM)	0.0	0.0	0.03–0.05	0.0	0.0	0.02–0.05	0.0	0.0	0.03–0.05
Total P (μM)	0.8	0.3	0.5–1.1	1.6	1.0	0.6–2.2	0.9	0.3	0.5–1.1
P-PO_4_ (μM)	0.4	0.2	0.1–0.5	0.6	0.3	0.3–0.9	0.4	0.3	0.1–0.7
Cl^-^ (mg/L)	5.6	1.7	3.5–7.6	6.2	2.1	3.5–8.6	5.7	2.0	4.1–9.2
SO_4_ (mg/L)	0.5	0.3	0.1–0.9	0.7	0.4	0.2–1.2	0.3	0.5	0.0–1.0
SiO_2_ (mg/L)	26.3	4.8	20.1–29.7	29.0	4.1	23.6–32.9	18.8	8.5	13.1–29.9
Na (mg/L)	6.5	6.8	3.0–19.9	5.0	1.9	3.3–8.5	4.9	3.5	0.6–8.1
K (mg/L)	0.3	0.3	0.1–0.8	1.1	0.6	0.5–2.1	0.5	0.8	0.0–1.7
Ca (mg/L)	1.1	0.3	0.7–1.4	0.6	0.3	0.3–1.0	1.2	0.3	0.7–1.6
Mg (mg/L)	1.7	0.7	0.9–2.9	0.5	0.4	0.1–0.9	0.9	0.5	0.2–1.5
Chlorophyll a (mg/m^2^)	1.5	1.8	0.3–5.1	0.8	0.8	0.3–2.4	0.9	1.0	0.1–2.4

### Environmental variables and chlorophyll

Stream physicochemistry was measured at each end of the study reach following methods in Vázquez et al. [24] during the dry season of 2019. Mean canopy cover over the stream was estimated with the HabitApp mobile application by averaging canopy coverage of 10 photographs taken with a leveled cell phone at points separated ~10 m along each study reach. The channel and riparian zone slopes were measured using a clinometer. Temperature, dissolved oxygen, and electrical conductivity were determined *in situ* using an YSI multi-meter (Mod. 85), and a potentiometer (Barnant Mod. 20) was used to measure pH. Water samples were collected in 1 L glass bottles at each end of the study reach. Samples were transported to the laboratory at 4°C and filtered through Whatman GF/C filters for analysis. In the laboratory, the following variables were measured using spectrophotometric techniques [25]: total suspended solids (TSS, dry weight), alkalinity (Alk, as CaCO_3_, phenolphthalein), ammonium (N-NH_4_^+^, Nessler), nitrate (N-NO_3_^-^, brucine), nitrite (N-NO_2_^-^, diazotization), chlorides (Cl-, titration with AgNO_3_), sulfates (SO_4_, turbidimetric technique) and silica (SiO_2_, molybdate). Samples were collected in 250 ml glass bottles for measuring total phosphorus (TP, persulfate digestion, and ascorbic acid) and reactive phosphorus (RP, ascorbic acid). Calcium (Ca^2+^) and magnesium (Mg^2+^) were measured using an atomic absorption spectrophotometer (Varian Mod. AA240FS) and sodium (Na^+^) and potassium (K^+^) were measured with a flame photometer (Corning Mod. 410).

Chlorophyll *a* concentration was measured at each study reach by collecting three rocks covered with biofilm and placing them in 90% methanol. Rocks were kept in the dark and refrigerated for 24 h before analysis. The chlorophyll *a* concentration was measured spectrophotometrically and its concentration was expressed in mg per m^-2^ [26].

### Resources and consumers

Resources consisted of terrestrial plant tissue and aquatic biofilms. Plant tissue consisted of freshly fallen senescent leaves from outside the stream, riparian trees (i.e., green leaves), fern leaves, and pasture leaves. Samples were labeled and transported to the laboratory in plastic bags for processing. Biofilm was scraped from the exposed side of four or five rocks, using a small brush and tray. We collected two to three samples of biofilm per stream. Scraped material was moved into glass containers and transported to the laboratory. Consumers consisted of 10 aquatic taxa from all major trophic guilds that were found in most streams (Table 2). Organisms were collected with aid of kick nets by sampling the stream substrate along the study reach. All organisms were placed in containers with water, maintained in cool conditions, and transported alive to the laboratory. Trophic guilds were assigned following Merritt et al. [27].

**Table 2:** Stable isotope signatures for resources and consumers per land use. Values are isotopic means and standard deviations (sd) of the number of samples (n) collected for each category. Trophic guilds are detritivores (D), herbivores (H), omnivores (O), and carnivores (C).

	Order or		Forest					Coffee Plantation					Pasture				
Taxa (guild)	common name		δ^13^C	sd	δ^15^N	sd	n	δ^13^C	sd	δ^15^N	sd	n	δ^13^C	sd	δ^15^N	sd	n
Biofilm			-24.8	9.7	-1.1	9.2	28	-28.2	1.9	2.5	3.0	18	-26.1	1.5	1.7	2.6	15
Leaf litter			-32.3	1.9	-1.9	2.2	28	-31.5	2.0	-0.5	2.5	18	-28.5	4.2	-1.9	2.0	15
Tipulidae	Diptera	D	-27.5	0.7	0.7	1.8	9	-27.4	1.2	2.4	3.4	2	-27.4	0.8	0.0	1.4	5
Calamoceratidae	Trichoptera	D	-27.5	0.9	2.6	2.1	15	-27.8	1.0	3.2	1.5	3	-28.3	1.2	0.8	1.2	7
Decapoda	Shrimp	D	-26.0	1.4	4.4	3.4	16	-26.9	2.1	5.3	2.5	8	-24.7	3.0	3.3	2.7	8
Tadpole		H	-26.9	2.1	4.6	2.8	21	-27.3	1.5	4.5	2.7	10	-27.7	3.5	4.3	2.0	10
Hydropsychidae	Trichoptera	O	-26.4	1.7	3.5	2.8	13	-25.5	2.3	6.3	2.3	5	-26.6	0.9	1.8	2.7	8
Poecilidae	Fish	O	-26.1	1.2	6.8	2.1	9	-26.1	2.2	7.5	2.3	8	-27.9	1.4	7.4	0.8	2
Libellulidae	Odonata	C	-25.9	1.8	4.5	2.4	25	-26.2	1.6	5.7	1.9	11	-25.6	2.9	3.5	2.1	11
Perlidae	Plecoptera	C	-26.5	1.6	4.1	2.4	18	-26.2	1.5	4.7	2.4	9	-24.6	1.8	4.7	2.0	9
Belostomatidae	Hemiptera	C	-25.8	1.3	4.0	2.6	16	-26.3	1.7	4.6	3.0	10	-26.6	3.1	2.9	2.2	7
Megaloptera	Megaloptera	C	-26.1	1.3	4.4	2.8	12	-25.8	1.6	4.4	4.1	9	-26.1	0.4	3.9	2.9	4

All samples were transported to the laboratory for processing and drying at 70°C for 48 hours. Leaves were placed in the oven in labeled paper bags, biofilm samples were left in their glass jars and oven-dried. Consumers were kept alive for 24 h at room temperature to allow for gut clearance, and were sorted by group and oven-dried for 48 h. Samples were ground to a fine powder, either manually or using a ball mill (Spex 8000D Mixer/Mill), for microcapsule preparation. All C and N isotope analyses were conducted at the Center for Applied Isotope Studies, the University of Georgia. Ratios of ^13^C/^12^C and ^15^N/^14^N are expressed in δ notation as the relative difference (‰) between the samples and international standards (Pee Dee Belemnite for C and air for N).

Resources and consumers were sampled during the dry and wet seasons of 2019. We did not assess seasonality, but expected a limited effect on isotopic signals. Seasonality in our study region affects the amount of resources available in streams, but not the types of resources [18] or composition of macroinvertebrate assemblages [28].

### Stable isotope mixing model

The proportion of terrestrial organic matter (allochthony) on consumers was estimated individually for each taxa belonging to four consumer guilds (i.e., herbivore, detritivore, omnivore, and carnivore) using a system of two linked Bayesian models. The first model was used for the determination of algal stable isotope ratios. The second model was used to determine isotopic mixing.

In our model to determine algal isotope ratios, we estimated the δ13C and δ15N of the algal component of biofilm by removing the contribution of terrestrial sources (e.g., leaf litter). While various techniques have been proposed to isolate algae in biofilms [29], our approach has the goal of determining the degree of allochthony in the system, not the specific algal isotope signals. To delimit the possible range in algal values, we used as informative priors the mean and standard deviation of 35 previous measurements of algae in tropical streams (S1 Table in S1 Appendix). Published algal information is only used to delimit possible algal isotopic values. Similar approaches have been found informative in studies of lake food webs [30–32].

We estimated isotopic ratios of algae (δ^13^C and δ^15^N) and corrected isotopic ratios of leaf litter fitting a mixing model for two sources within a hierarchical Bayesian framework [33, 34]:

$$\delta_{P}∿Ɲ(\delta_{A}\times (1-\Phi_{T})+\delta_{T}\times {\Phi_{T},\sigma }_{})$$ (1)

where δ_P_ is biofilm stable isotopic signature (i.e. δ^13^C or δ^15^N), δ_A_ is the algal stable isotopic signature, and *Φ*_*T*_ is a terrestrial fraction on biofilm, and δ_T_ is for stable isotope signature of allochthonous matter (i.e., leaf litter). We were most interested in the values of δ_A_ for each land use; given that there were 3 categories (*f*, *c*, and *p*, for forest, coffee plantation, and pasture, respectively) [34]. We set forest category as baseline (*δ*_*f*_), such that its prior probability was

$$\delta_{A}∼Ɲ(\delta_{f}+\beta_{c}+\beta_{P},\sigma_{\delta })$$ (2)

where *δ*_*f*_ for δ^13^C had a prior distribution of *δ*^13^*C*~Ɲ(−23.72, 4.10), and for δ^15^N had a prior distribution of *δ*^15^*N*~Ɲ(4.10, 3.13). Terms β_c_ and β_p_ correspond to group indicators for coffee plantations (c) and pasture (p), respectively. Terms for each land use category other than forest had a prior normalized distribution with a mean of 0 and a standard deviation (sd) of 1. As with δ_A_, we set forest category as a baseline for *Φ*_*T*_ and used α_c_ and α_p_ as terms for coffee plantations and pasture respectively, such that

$$\Phi_{Tj}=\Phi_{f}-\alpha_{c}-\alpha_{p}$$ (3)

where *Φ*_*f*_ had a prior distribution of *Φ*_*f*_~*U*(0,1), where 0 would be 100% of the autochthonous composition and 1 a 100% of the allochthonous composition. Terms α_c_ and α_p_ had a prior normalized distribution with a mean of 0 and a sd of 1.

The second model was a mixing model. After estimating the isotopic ratio of algae using the model described above, these values were passed into a mixing model of the form:

$$\delta_{t,c}=\delta_{t,1:s}(\Delta \delta \times L)\circ {\Phi_{1:s}}_{}$$ (4)

where *δ*_*t*,*c*_ is the isotopic ratio of each freshwater consumer (c) for each tracer (t), *δ*_*t*,1:*s*_ is the vector of isotopic ratios for each of its potential carbon sources (1:s), *Δδ* is the trophic fractionation factor by each tracer (i.e., *Δδ*^13^*C* and *Δδ*^15^*N*), L is the trophic level for each consumer, and *Φ* is a vector of source proportions (drawn from a Dirichlet distribution with alpha set to a vector of ones). The symbol ∘ represents the scalar product. δ_t, 1:s_ had a prior normalized distribution with mean and sd values of δ^13^C or δ^15^N of our leaf litter samples, and estimated mean and sd for algae by each land use (S2 Table in S1 Appendix). Prior probabilities for *Δδ* were *Δδ*^13^*C*~Ɲ(0.39, 1.14) and *Δδ*^15^*N*~Ɲ(3.4, 0.9) [35]. L had a uniform distribution with prior probability between 0 and 10.

We fit these models by simulating the posterior parameter distributions using the program Stan [36] using the rstan package in R [37]. Stan uses a Markov chain Monte Carlo (MCMC) method to simulate posterior distributions. MCMC sampling of posteriors was performed with four chains and 5000 iterations by chain, after burn-in. We visually checked the chains for convergence and that of the scale reduction factor, $\hat{R}<1.1$, for all parameters.

### Statistical analyses

Tendencies of variation in physicochemical variables among streams were assessed using a Principal Component Analysis (PCA). Variables were log_10_ (x + 1) transformed to normalize the data, except for pH [38]. The PCA used centered and standardized data. Analyzes were carried out with the MVSP program [39]. Depending on the normality and homoscedasticity of data, we performed ANOVAS or Kruskal-Wallis One Way Analysis of Variance on Ranks to assess physicochemical differences among streams.

## Results

### Land use and stream physicochemical characteristics

Stream physicochemistry during the dry season rendered few differences between streams (Table 1). There were no significant differences among land uses in most of their physical and chemical characteristics. Mean water temperature in coffee plantations was higher (17.1°C) than in forest and pasture streams (14.6°C). All streams were well-oxygenated (> 5.8 mg/L). Water conductivity in forest was higher than that of coffee and pasture streams (Table 1). The pH fluctuated from 6.6 to 7.0 in all streams. Significantly higher concentrations of Total Suspended Solids (TSS) were recorded in coffee plantation streams than in forests (H = 6.5, P <0.03). N-NH_4_ concentrations were significantly higher in forests and coffee plantations (2.7–3.3 μM) than in pastures (0.1 μM) (H = 6.3, P <0.03). The concentration of N-NO_3_ was higher in the forests than in the coffee plantations and pastures. The TP was recorded in higher concentrations in coffee plantations than in forests and pastures (Table 1). Chlorophyll *a* concentration was variable, but not significantly different among land uses (Table 1).

**Fig 2:**
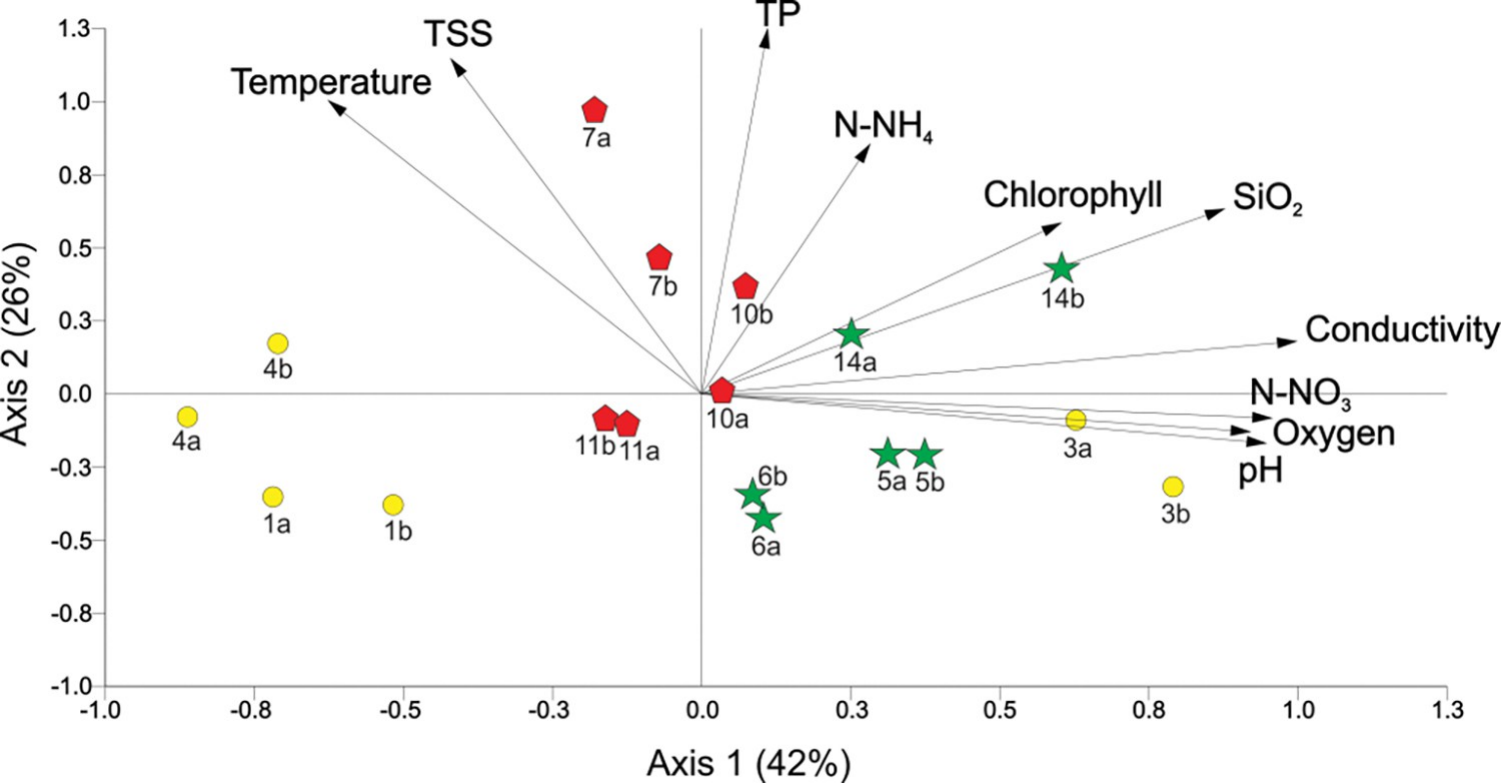
Principal Component Analysis (PCA) of study sites based on water physicochemistry. Variables were measured at each end of the study reach and two points are representing each stream (points a and b). Numbers correspond to stream names in Table 2 and symbols to land uses, forest (green stars), coffee plantation (red pentagons), and pastures (yellow circles).

Streams draining each land use had similar physicochemical characteristics (Fig 2). PCA analysis grouped all streams by land use, except a single stream (Fig 2). Axes 1 and 2 explained 42% and 26% of the total variance. Forest streams and one of our pasture streams grouped in the quadrants on the right side of the biplot, displayed the highest conductivities and concentrations of N-NO_3_, oxygen, pH, SiO_2_, and chlorophyll *a*. In contrast, the other two pasture streams grouped in the quadrants on the left side of the biplot, showed low concentrations of all variables. The coffee plantation streams were grouped in the upper quadrants mainly associated with high TP, TSS, and temperature concentrations.

### Basal resources and consumers

**Fig 3:**
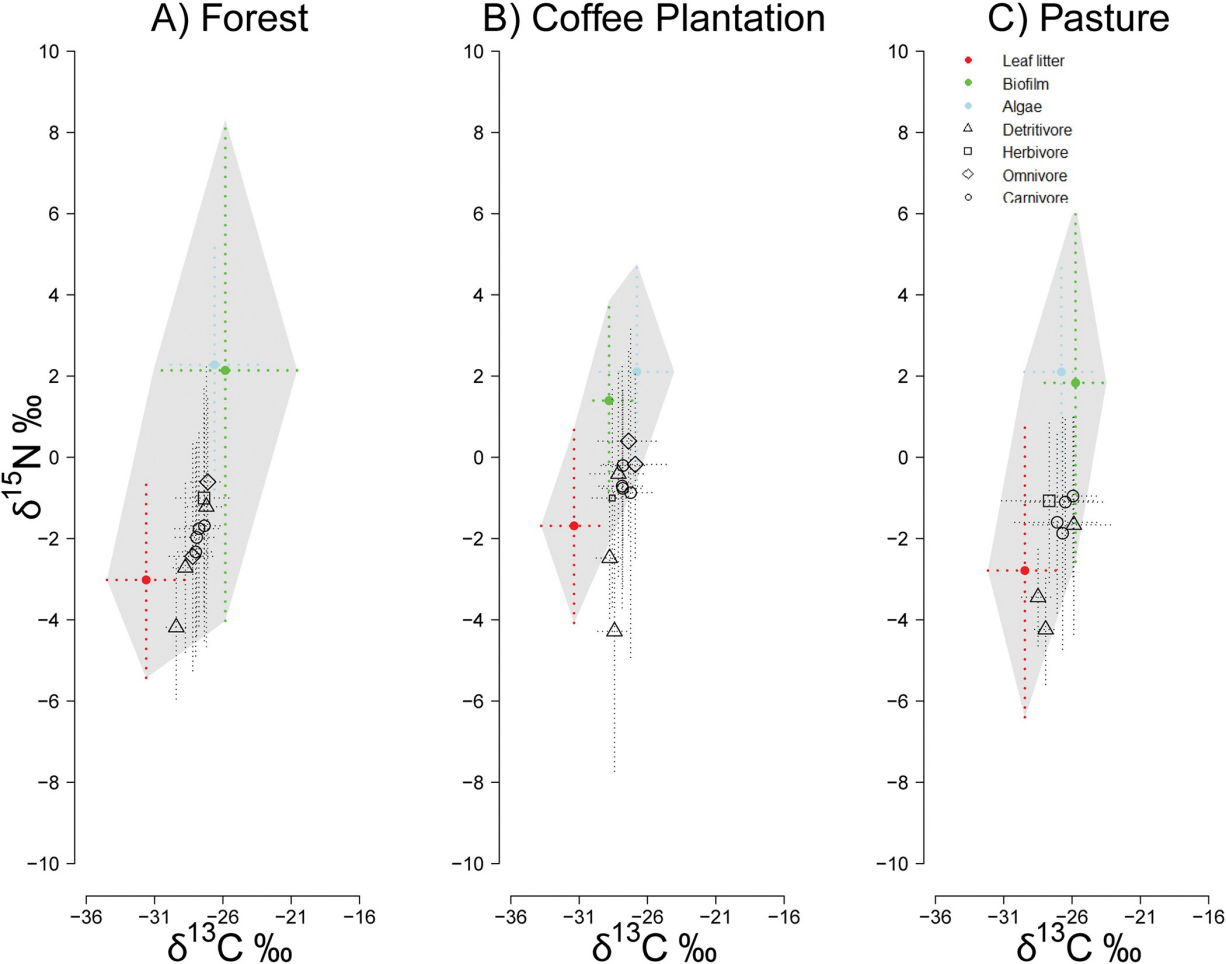
Biplots of resources and consumers under three land uses in the upper La Antigua, Mexico. Error bars indicated 1 standard deviation. Resources are solid circles: Algae (blue), biofilm (green), and leaf litter (red). Consumers are corrected to account for trophic fractionation and are represented by open symbols: detritivores (triangles), herbivores (squares), omnivores (diamonds), and carnivores (circles).

Biofilm and leaf litter were the basal resources sampled at all streams. Biofilm had similar isotopic values for C in forest than in pasture, but was highest in coffee plantations, while N was highest in coffee plantations. Leaf litter had similar C signals in forest and coffee plantations, and lower in pastures, while N was lowest in coffee plantations (Table 2). The isotopic position of biofilm was always in between leaf litter and algae, as it combines both resources (Fig 3). To address our main objective of determining the degree of allochthony on stream food webs, we focused on algal vs. leaf litter C isotopic signatures. We found little overlap between algae and leaf litter C signals in forest and coffee plantation streams; with pasture streams showing a larger degree of C overlap (S3 Fig in S1 Appendix).

Not all consumer taxa were abundant in all streams, but we collected enough samples of all trophic guilds (Table 2). Herbivores had similar C and N signals among land uses, average C ranged from -26.9 to -27.7‰ and N ranged from 4.3 to 4.6‰. Detritivores also had similar C among land uses (range: -26.0 to -28.3‰), but their N was lowest in pastures (range: 0 to 3.3‰). Omnivores also had a narrow range in their C signals (range: -25.5 to -27.9‰) and their N was variable with a larger range in pasture streams (range in pasture: 1.8 to 7.4‰). Carnivores had C signals that ranged from -24.6 to -26.5‰, and their lowest N signals occurred in pasture (range: 2.0 to 2.9‰; Table 2).

Bi-plots for stable isotopes, after accounting for trophic fractionation, showed that all trophic guilds had isotopic signals similar to leaf litter rather than algae in all land uses (Fig 3). As could be expected, detritivores are located close to leaf litter, while omnivores and carnivores are closer to biofilm in all land uses. Overall, consumers fall within the region covered by resource variability (Fig 3).

All trophic guilds show high levels of allochthony (Table 3). Consumers in streams draining the three land uses assimilated on average 34% of their C from leaf litter relative to only 20% from algae (means in Table 3).

**Table 3:** Contribution of resources to consumer isotopic signals. Trophic guilds are detritivores (D), herbivores (H), omnivores (O), and carnivores (C).

Trophic Guild		Leaf litter				Algae				Trophic Level	Trophic Enrichment Factor	
		Mode	Mean	CI (2.5–97.5)		Mode	Mean	CI (2.5–97.5)		Mean	Δ^13^C	Δ^15^N
**Forest**												
Tipulidae	D	0.033	0.471	0.005	0.973	0.213	0.070	0.002	0.249	1.979	0.967	2.466
Calamoceratidae	D	0.027	0.276	0.002	0.970	0.107	0.075	0.001	0.556	1.962	0.632	2.689
Decapoda	D	0.170	0.159	0.005	0.721	0.120	0.274	0.003	0.895	1.997	0.597	2.821
Tadpole	H	0.065	0.151	0.005	0.831	0.123	0.269	0.002	0.914	1.956	0.238	2.839
Hydropsychidae	O	0.025	0.360	0.004	0.927	0.349	0.139	0.005	0.522	2.171	0.852	2.733
Fish	O	0.036	0.195	0.003	0.917	0.256	0.302	0.003	0.934	2.456	0.394	3.030
Odonata	C	0.041	0.281	0.002	0.964	0.126	0.155	0.002	0.914	2.226	0.830	2.823
Plecoptera	C	0.557	0.259	0.002	0.967	0.025	0.139	0.002	0.925	2.116	0.676	2.864
Belostomatidae	C	0.802	0.358	0.002	0.952	0.124	0.129	0.003	0.822	2.304	0.934	2.752
Megaloptera	C	0.661	0.243	0.004	0.914	0.044	0.207	0.004	0.884	2.146	0.599	2.853
**Coffee Plantations**												
Tipulidae	D	0.596	0.365	0.012	0.870	0.326	0.265	0.009	0.765	2.277	0.425	2.918
Calamoceratidae	D	0.405	0.391	0.009	0.915	0.166	0.209	0.006	0.750	2.043	0.490	2.786
Decapoda	D	0.222	0.311	0.006	0.917	0.026	0.214	0.004	0.829	1.952	0.606	2.914
Tadpole	H	0.237	0.358	0.005	0.955	0.331	0.149	0.003	0.818	1.973	0.653	2.793
Hydropsychidae	O	0.657	0.266	0.008	0.822	0.004	0.333	0.008	0.860	2.154	0.659	2.992
Fish	O	0.006	0.241	0.005	0.890	0.551	0.313	0.006	0.904	2.314	0.563	3.064
Odonata	C	0.157	0.285	0.003	0.930	0.205	0.201	0.004	0.895	2.050	0.792	2.893
Plecoptera	C	0.613	0.307	0.004	0.920	0.081	0.202	0.004	0.858	1.986	0.821	2.763
Belostomatidae	C	0.570	0.295	0.005	0.916	0.217	0.203	0.005	0.831	1.917	0.792	2.777
Megaloptera	C	0.173	0.266	0.005	0.912	0.455	0.275	0.005	0.893	1.934	0.752	2.729
**Pasture**												
Tipulidae	D	0.570	0.617	0.027	0.964	0.093	0.111	0.003	0.418	1.725	0.323	2.465
Calamoceratidae	D	0.586	0.676	0.056	0.967	0.209	0.090	0.002	0.352	1.658	0.098	2.532
Decapoda	D	0.026	0.352	0.010	0.902	0.141	0.244	0.006	0.776	1.821	0.650	2.701
Tadpole	H	0.288	0.372	0.016	0.905	0.409	0.201	0.004	0.734	1.848	-0.024	2.925
Hydropsychidae	O	0.022	0.574	0.016	0.933	0.238	0.130	0.004	0.426	1.730	0.441	2.624
Fish	O	0.300	0.351	0.012	0.872	0.004	0.279	0.008	0.807	2.747	0.044	3.094
Odonata	C	0.362	0.322	0.014	0.799	0.105	0.223	0.005	0.721	1.692	0.510	2.731
Plecoptera	C	0.551	0.361	0.007	0.864	0.238	0.242	0.007	0.721	1.940	0.666	2.938
Belostomatidae	C	0.071	0.289	0.016	0.841	0.456	0.206	0.005	0.765	1.698	0.299	2.661
Megaloptera	C	0.040	0.354	0.012	0.911	0.095	0.205	0.003	0.779	2.026	0.290	2.837

**Fig 4:**
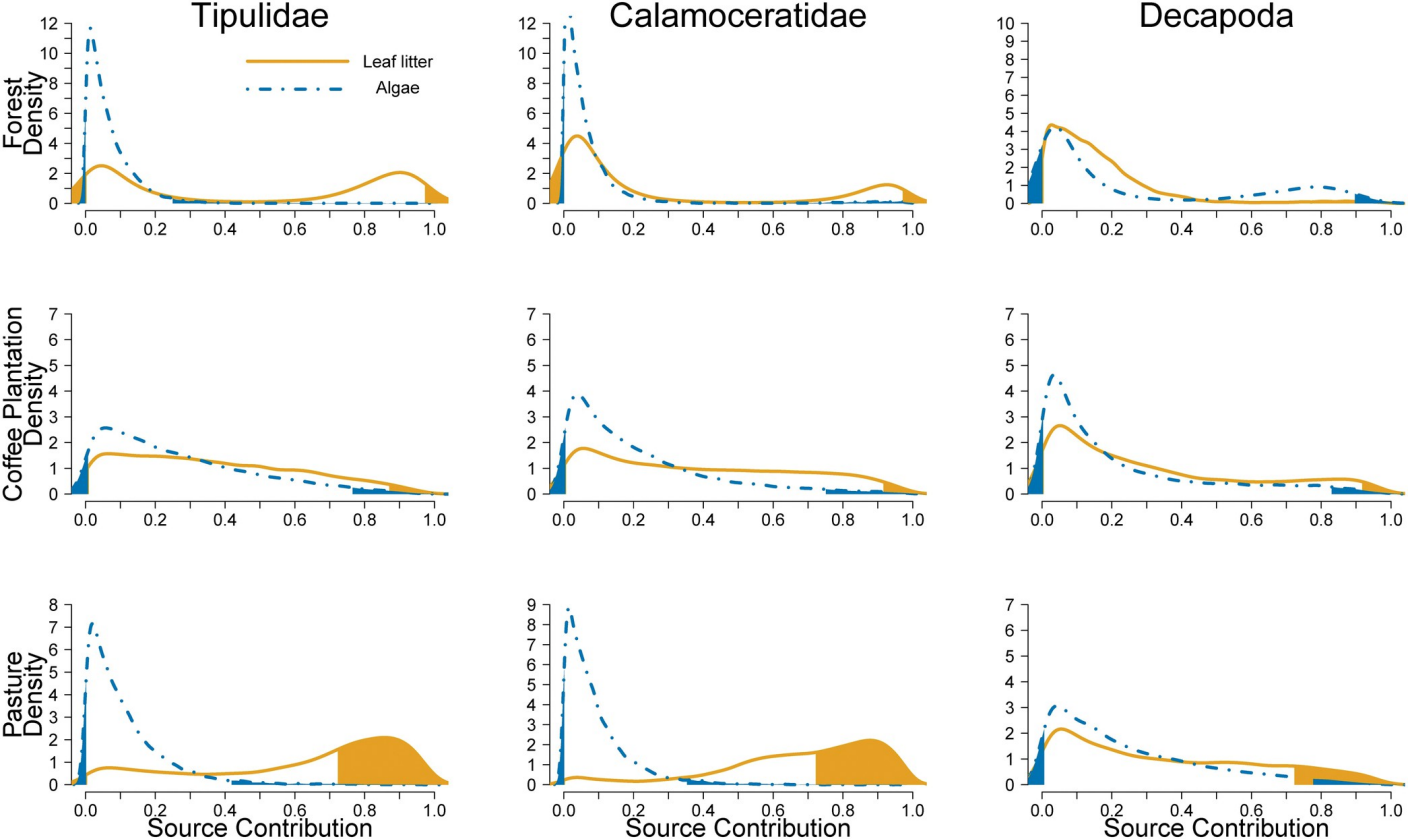
Resource consumption by detritivores. Posterior probability density of proportional source contributions by land use. The unshaded region corresponds to the 95% credible interval.

**Fig 5:**
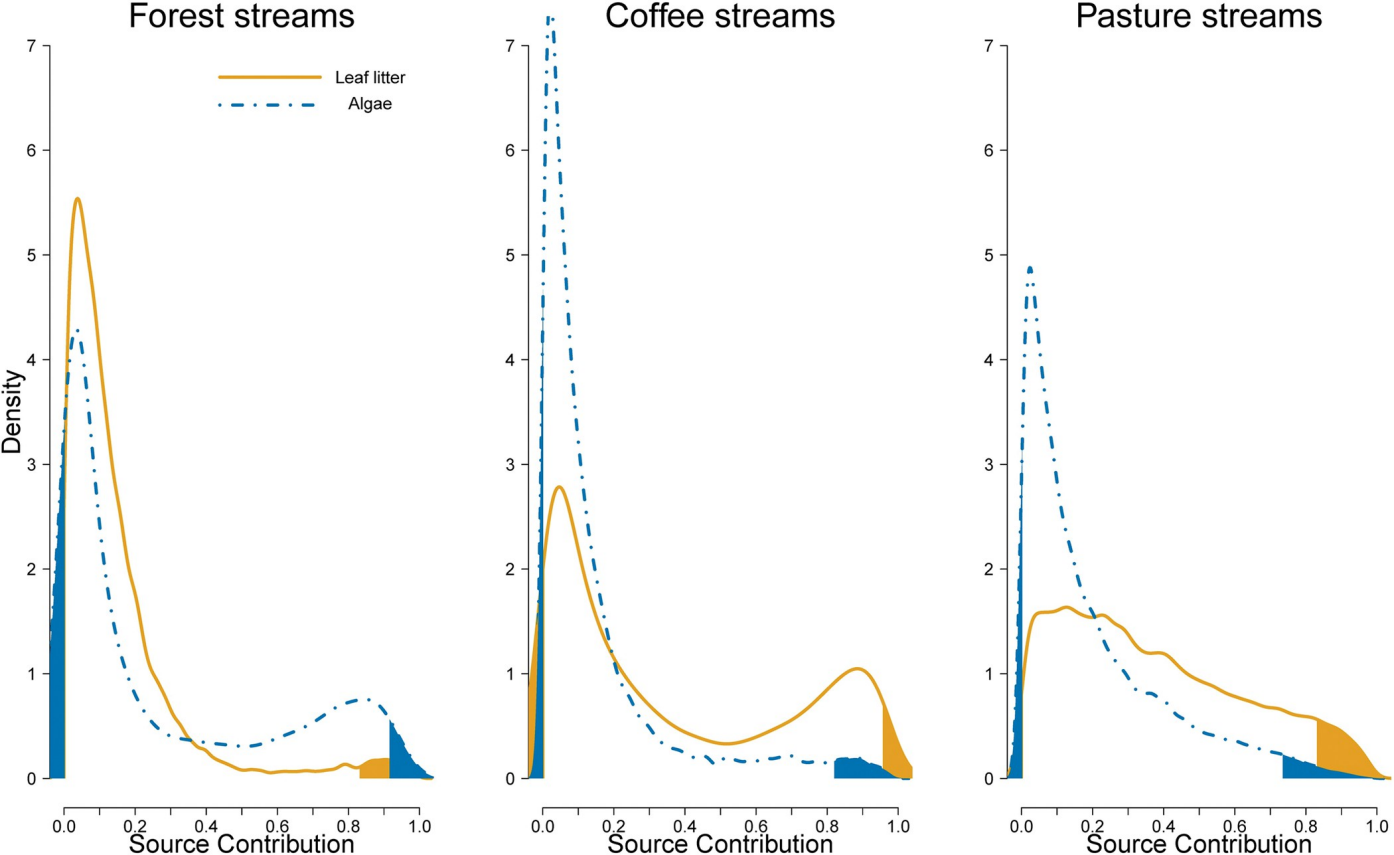
Resource consumption by herbivores (tadpoles). Posterior probability density of proportional source contributions by land use. The unshaded region corresponds to the 95% credible interval.

**Fig 6:**
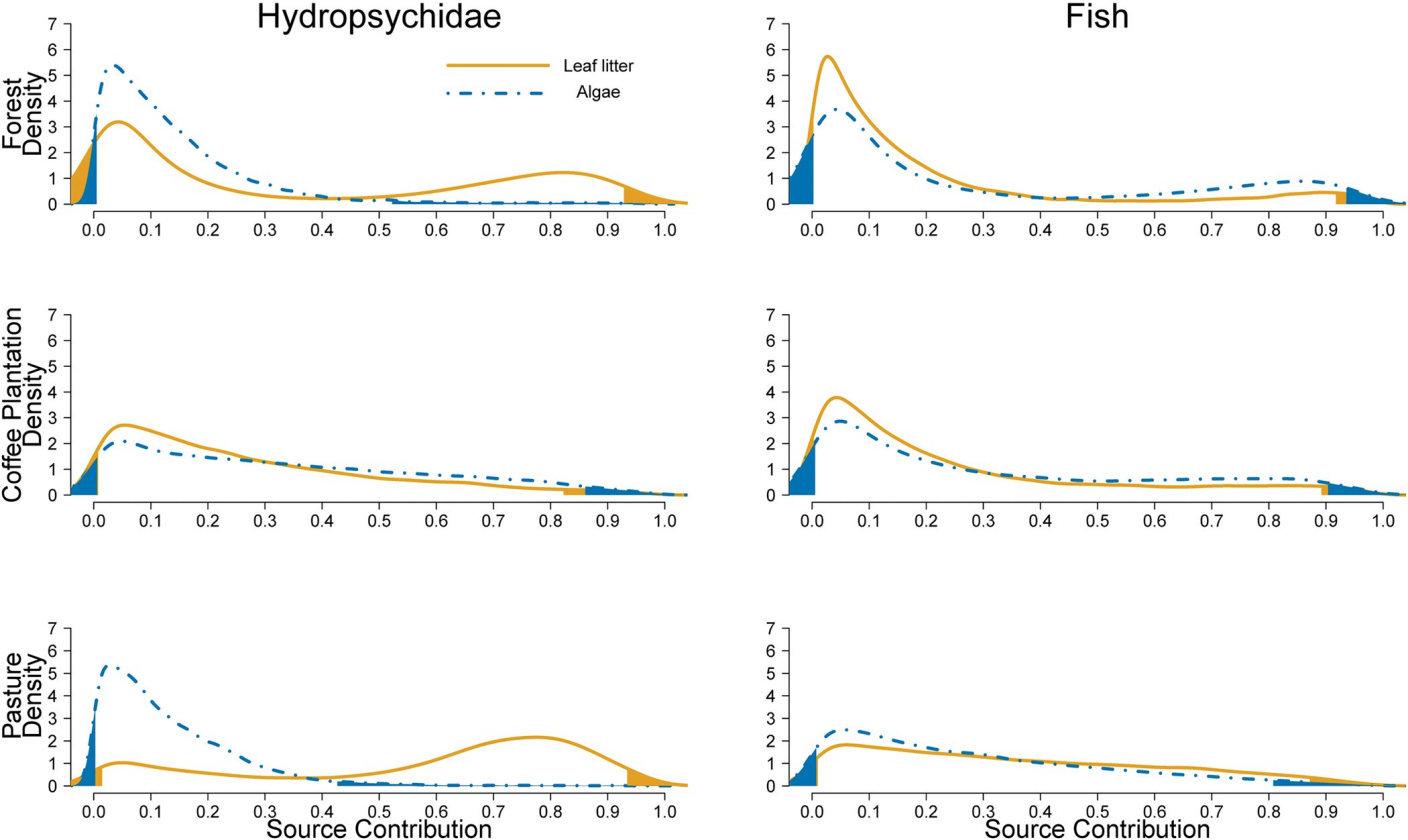
Resource consumption by omnivores. Posterior probability density of proportional source contributions by land use. The unshaded region corresponds to the 95% credible interval.

**Fig 7:**
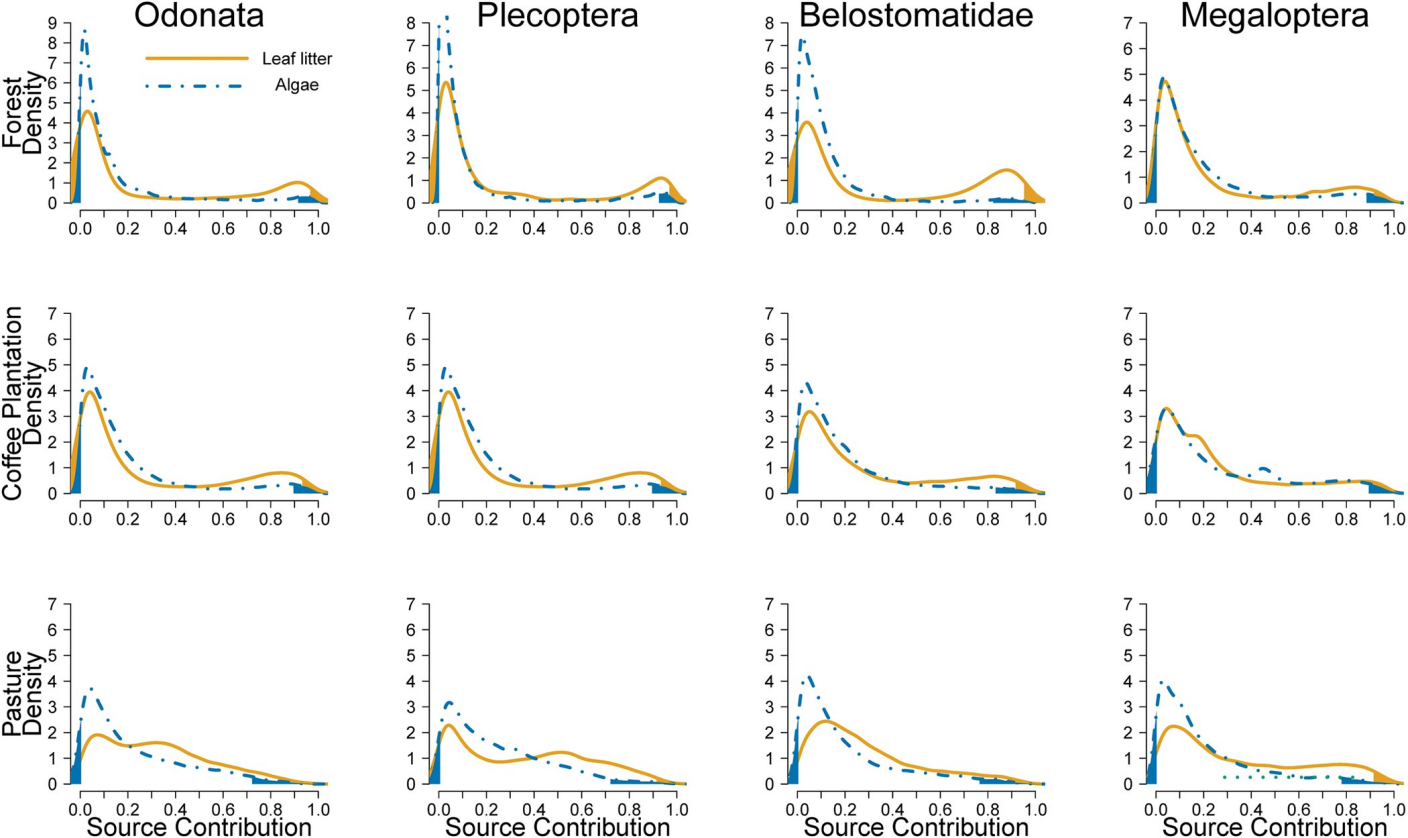
Resource consumption by carnivores. Posterior probability density of proportional source contributions by land use. The unshaded region corresponds to the 95% credible interval.

The degree of allochthony on stream food webs was determined by comparing assimilation patterns of algal C vs. leaf litter C. Detritivorous consumers showed the expected pattern of relying mostly on leaf litter and only decapods show some degree of algal consumption in forest streams (Fig 4). Herbivores in forest had low levels of leaf litter assimilation, but leaf litter was their main resource in coffee plantations and pastures (Fig 5). Omnivores consumed mostly leaf litter or had similar proportions of algae and leaf litter (Fig 6). Carnivores showed a reliance on leaf litter C, with some taxa also using algal C as an important resource (e.g., Megaloptera; Fig 7).

## Discussion

Cloud forest streams in La Antigua watershed have food webs that rely primarily on allochthonous C, regardless of the land use surrounding them. Our findings indicate that food webs in forest, coffee plantations, and pasture streams have allochthonous and autochthonous resources available, but consumers mostly use C derived from allochthonous sources. This contradicts our initial hypothesis that stated that only forest streams would rely on allochthonous C, while pasture streams were expected to use mostly autochthonous C. Remaining patches of vegetation in riparian zones and high runoff in these rainy ecosystems potentially explain the persistence of terrestrial C. Our findings indicate that cloud forest streams have high levels of allochthony, based on the assimilation of C from terrestrial sources (following [30]).

The amount of allochthony in stream food webs is expected to be the result of land use cover and stream size [40]. Small headwater streams draining forest receive ample inputs of leaf litter and their narrow channels do not create gaps in the canopy, limiting the amount of sunlight that reaches the stream bottom [40, 41]. The role of allochthonous C in fueling food webs in low order streams is well-established, in particular for temperate region streams [2, 3]. Multiple trophic levels in those streams are dependent on inputs of leaf litter from riparian vegetation [2]. In contrast, studies in tropical streams surrounded by forest have produced mixed evidence [5]. While some have documented similar degrees of allochthony as in temperate regions [42], others propose a larger role for algal C [4, 43]. Recent studies using a combination of approaches (e.g., stable isotopes and stream metabolism) has shown that some tropical stream food webs rely mostly on allochthonous C, even when algal C is available [7]. Our study provides further evidence for the importance of allochthonous C in cloud forest streams, where food webs are strongly linked to C from riparian zones.

Streams draining natural areas of tropical cloud forest can be expected to rely on allochthonous resources. Our previous studies in this cloud forest area provide evidence for a high degree of allochthony in stream food webs. Using gut content analysis of dominant aquatic insects, García et al. [16] reported a predominance of plant tissue and amorphous detritus in most benthic consumers. The study found a range of 31–99% of aquatic insect gut contents composed of allochthonous material in forest streams. Similarly, gut contents in a pasture stream without riparian vegetation were found to have up to 82% allochthonous material. Gut content analysis has the limitation of focusing on ingested, rather than assimilated energy. However, our analysis using isotopes support those findings, suggesting that allochthony is dominant in forest and pasture streams in cloud forest. Further evidence for the importance of allochthonous resources comes from insect scrappers. Scrappers inhabit hard substrates in streams and are expected to consume mostly diatoms. However, this functional group was found to be a dominant component of macroinvertebrate assemblages in cloud forest streams [28]. Their gut contents often contained high proportions of allochthonous material in both forest and pasture streams [16].

Deforestation of riparian vegetation and land use conversion from forest to agriculture alter many environmental characteristics and the functioning of stream ecosystems [8]. Removal of riparian vegetation often changes resource availability for stream food webs, decreasing inputs of leaf litter by replacing riparian trees with crops or pastures [44]. At the same time, a lack of arboreal vegetation increases solar radiation in the stream channel and enhances algal biomass and production [45]. Macroinvertebrate assemblages often change their composition; losing groups specialized in consuming leaf litter and gaining scrappers and herbivorous taxa. This pattern has been described for temperate [46, 47] and tropical streams [48, 49]. Our study shows that consumers continue relying on allochthonous C, even when coffee plantations and pastures provide reduced riparian cover relative to a forest. In our cloud forest study area, forest conversion to agriculture and pasture is a dominant trend [19] with consequent negative impacts on water physicochemistry [18] and macroinvertebrate assemblage composition and structure [28].

### Land use and cloud forest streams

Land use had a clear effect on the physical and chemical characteristics of our study cloud forest streams. Our sampling of stream physicochemistry reflected conditions under reduced flow and few floods. Important differences were evident in the total suspended solids and ammonia concentrations in streams, which were more abundant in coffee plantation or pasture streams than in forest streams. Similar results have been reported in field studies and simulated analysis using SWAT models showing that land use increases TSS and nutrient concentrations in streams surrounded by agricultural lands compared to forests [50, 51]. Our results evidenced weaker land use impacts on algal biomass than those reported for the same area in previous studies [18]. Algal biomass (i.e., chlorophyll concentrations) was similar among streams in our study, even slightly higher in forest. However, Vázquez et al. [18] found consistently higher algal biomass in pasture streams than in forest. The difference could be related to the much higher concentration of suspended solids that we found in our study.

The predominance of food web allochthony under all land uses suggests that leaf litter might not be the only source of terrestrial C for streams. Forest streams receive abundant direct inputs of C from riparian vegetation, via vertical inputs of leaves [28]. Coffee plantations maintain riparian cover, even when it might be reduced or replaced by crop plants, and leaves inputs could be expected to be abundant. Our pasture streams maintained patches of riparian vegetation, which included scattered and isolated riparian trees and thin strips of riparian forest. However, in this wet and rainy environment, water runoff into the stream channel (superficial and as local groundwater) is abundant, in particular during the wet season [18], and streams can receive large amounts of fine particles from the stream banks and adjacent watershed. Allochthonous C could enter streams as fine particles during rain events, thus supplying food webs with organic matter. Scattered tree cover is often present in pastures, either close to the stream or as shade for cattle. This vegetation along with pastures could be an important source of C that is captured by the biofilm in streams and moves into food webs as scrappers and other consumers feed on the biofilms over rocks.

Our study documented, for the first time, the impact of land use on the C basis of stream food webs in a tropical cloud forest. The predominance of allochthony suggests that deforested watersheds maintain enough sources of allochthonous C to fuel stream food webs. The abundant precipitation is expected to be responsible for moving allochthonous C to the stream, even in open pasture areas, where stream consumers use it. In our study region, conversion of forest into other land uses results in significant changes in stream water physicochemistry, in particular increasing sediment and nutrient inputs into streams (our study; [18]). Biotic assemblages, especially macroinvertebrates, become simplified, as they lose sensitive taxa [28, 52]. Ecosystem processes are also affected by the land use surrounding the stream. For example, leaf-litter decomposition rates increase as forest cover decreases, potentially the result of increases in nutrient levels and other disturbances [53]. Thus, while food webs remain based on allochthonous resources, multiple other ecosystem components change with land use change.

## Supporting information

S1 Appendix Supporting information includes: (S1 Table) Records of isotopic ratios for algae in tropical streams; (S2 Table) mean and standard deviation (SD) of basal resources per land use; and (S3 Fig) resource overlap between algal C and N (blue lines) and leaf litter C and N (orange) for each land use. (DOCX)
