# Supplementary material for: Stream food webs in tropical mountains rely on allochthonous carbon regardless of land use
Source: PLoS One. 2023 Dec 15;18(12):e0295738. doi: 10.1371/journal.pone.0295738 (PMC10723698; doi:10.1371/journal.pone.0295738)
Supplement: S1 Appendix — (DOCX) [file pone.0295738.s001.docx]

| **Table S1**. Records of isotopic ratios for algae in tropical streams. | | | | |
| --- | --- | --- | --- | --- |
|  |  |  |  |  |
| **Item** | **δ^13^C** | **δ^15^N** | **Source** | **Country** |
| Microalgae | -25.60 | 2.50 | Brito et al. (2006) | Brazil |
| Filamentous | -25.10 | 1.40 | Brito et al. (2006) | Brazil |
| Microalgae | -21.60 | 2.30 | Brito et al. (2006) | Brazil |
| Microalgae | -21.30 | 2.30 | Brito et al. (2006) | Brazil |
| Microalgae | -19.00 | 1.40 | Brito et al. (2006) | Brazil |
| Macroalgae | -32.72 | 4.37 | Ceneviva‐Bastos et al. (2017) | Brazil |
| Macroalgae | -27.92 | 6.86 | Ceneviva‐Bastos et al. (2017) | Brazil |
| Macroalgae | -21.70 | 10.53 | Ceneviva‐Bastos et al. (2017) | Brazil |
| Macroalgae | -19.25 | 8.81 | Ceneviva‐Bastos et al. (2017) | Brazil |
| Macroalgae | -17.60 | 6.55 | Ceneviva‐Bastos et al. (2017) | Brazil |
| Macroalgae | -16.01 | 6.89 | Ceneviva‐Bastos et al. (2017) | Brazil |
| Filamentous | -22.70 | 7.70 | Coat et al. (2009) | Guadalupe |
| Spirogyra | -34.30 | 1.90 | Douglas et al. (2005) | Australia |
| Nostoc | -18.80 | -0.80 | Douglas et al. (2005) | Australia |
| Cyanobacteria | -21.70 | -0.90 | Dudgeon et al. (2010) | Hong Kong |
| Cyanobacteria | -20.00 | -1.50 | Dudgeon et al. (2010) | Hong Kong |
| Filamentous | -28.84 | 2.61 | Lau et al. (2009) | Hong Kong |
| Filamentous | -27.73 | 1.85 | Lau et al. (2009) | Hong Kong |
| Filamentous | -27.20 | 6.00 | Lau et al. (2009) | Hong Kong |
| Filamentous | -27.20 | 6.00 | Lau et al. (2009) | Hong Kong |
| Cyanobacteria | -24.60 | 0.27 | Lau et al. (2009) | Hong Kong |
| Cyanobacteria | -22.90 | 4.54 | Lau et al. (2009) | Hong Kong |
| Cyanobacteria | -20.53 | 5.97 | Lau et al. (2009) | Hong Kong |
| Cyanobacteria | -16.87 | 8.13 | Lau et al. (2009) | Hong Kong |
| Filamentous | -18.36 | 5.96 | March and Pringle (2003) | Puerto Rico |
| Filamentous | -18.24 | 1.40 | March and Pringle (2003) | Puerto Rico |
| Filamentous | -17.03 | 2.16 | March and Pringle (2003) | Puerto Rico |
| Macroalgae | -28.30 | 2.40 | Unpublished | Mexico |
| Filamentous | -24.70 | 3.60 | Unpublished | Mexico |
| Filamentous | -33.20 | 2.03 | Verburg et al. (2007) | Panama |
| Filamentous | -37.94 | 0.92 | Winemiller et al. (2011) | Belize |
| Filamentous | -25.84 | 4.32 | Winemiller et al. (2011) | Belize |
| Filamentous | -22.02 | 5.01 | Winemiller et al. (2011) | Belize |
| Filamentous | -14.24 | 6.87 | Winemiller et al. (2011) | Belize |

**References**

Brito EF, Moulton TP, De Souza ML, Bunn SE. Stable isotope analysis indicates microalgae as the predominant food source of fauna in a coastal forest stream, south‐east Brazil. Austral Ecol. 2006;31:623-633.

Ceneviva‐Bastos M, Montaña C G, Schalk CM, Camargo PB, Casatti L. Responses of aquatic food webs to the addition of structural complexity and basal resource diversity in degraded Neotropical streams. Austral Ecol. 2017;42:908-919.

Coat S, Monti D, Bouchon C, Lepoint G. Trophic relationships in a tropical stream food web assessed by stable isotope analysis. Freshw Biol. 2009;54:1028-1041.

Douglas MM, Bunn SE, Davies PM. River and wetland food webs in Australia’s wet–dry tropics: general principles and implications for management. Mar Freshw Res. 2005;56:329-342.

Dudgeon D, Cheung FK, Mantel SK. Foodweb structure in small streams: do we need different models for the tropics? J North Am Benthol Soc. 2010;29:395-412.

Lau DC, Leung KM, Dudgeon D. What does stable isotope analysis reveal about trophic relationships and the relative importance of allochthonous and autochthonous resources in tropical streams? A synthetic study from Hong Kong. Freshw Biol. 2009;54:127-141.

March JG, Pringle CM. Food web structure and basal resource utilization along a tropical island stream continuum, Puerto Rico. Biotropica. 2003;35:84-93.

Verburg P, Kilham SS, Pringle CM, Lips KR, Drake DL. A stable isotope study of a neotropical stream food web prior to the extirpation of its large amphibian community. J Trop Ecol. 2007;23:643-651.

Winemiller KO, Hoeinghaus DJ, Pease AA, Esselman PC, Honeycutt RL, Gbanaador D, Carrera E, Payne J. Stable isotope analysis reveals food web structure and watershed impacts along the fluvial gradient of a Mesoamerican coastal river. River Res Appl. 2011;27:791-803.

| **Table S2**. Mean and standard deviation (SD) of basal resources per land use. | | | | | | |
| --- | --- | --- | --- | --- | --- | --- |
|  |  |  |  |  |  |  |
|  |  | **δ^13^C** | |  | **δ^15^N** | |
|  | **Basal resource** | **mean** | **SD** |  | **mean** | **SD** |
| Forest | |  |  |  |  |  |
|  | Leaf litter | -32.33 | 1.87 |  | -1.95 | 2.16 |
|  | Periphyton | -24.80 | 9.67 |  | -1.08 | 9.15 |
|  | Algae | -23.65 | 2.41 |  | 2.37 | 1.83 |
| Coffee plantation | |  |  |  |  |  |
|  | Leaf litter | -31.52 | 1.96 |  | -0.47 | 3.00 |
|  | Periphyton | -28.18 | 1.92 |  | 2.48 | 2.46 |
|  | Algae | -23.64 | 2.35 |  | 2.20 | 1.72 |
| Pasture | |  |  |  |  |  |
|  | Leaf litter | -28.54 | 4.23 |  | -1.91 | 1.96 |
|  | Periphyton | -26.06 | 1.46 |  | 1.70 | 2.59 |
|  | Algae | -23.63 | 2.35 |  | 2.19 | 1.70 |
|  |  |  |  |  |  |  |


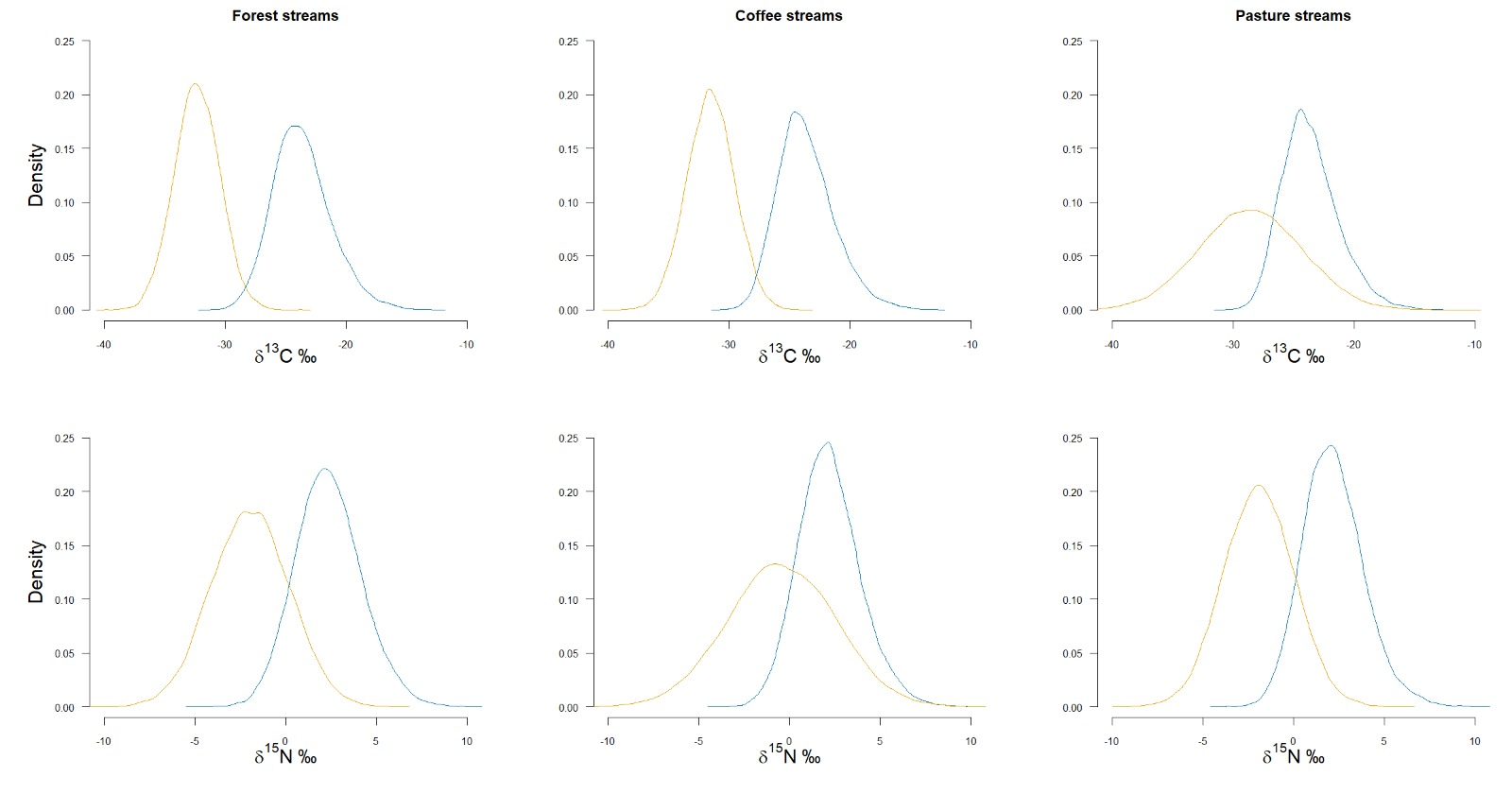


S3 Figure. Resource overlap between algal C and N (blue lines) and leaf litter C and N (orange) for each land use.
